# P-1236. Clinical Impact of Linezolid Therapeutic Drug Monitoring

**DOI:** 10.1093/ofid/ofaf695.1428

**Published:** 2026-01-11

**Authors:** Joseph Johnson, Elizabeth A Scruggs-Wodkowski, Robert Woods, Ronald E Kendall

**Affiliations:** VA Ann Arbor Healthcare System, Ann Arbor, Michigan; Veteran Affairs Ann Arbor Healthcare System; University of Michigan Medical School, Ann Arbor, Michigan; University of Michigan, Ann Arbor, Michigan; VA Ann Arbor Healthcare System, Ann Arbor, Michigan

## Abstract

**Background:**

Linezolid use is increasing in the outpatient setting to treat severe infections caused by Gram-positive bacteria, such as vancomycin-resistant enterococci (VRE) and methicillin-resistant Staphylococcus aureus (MRSA). Although FDA approved linezolid at a fixed dose of 600 mg twice daily, newer literature describes the importance of therapeutic drug monitoring (TDM) due to linezolid’s narrow therapeutic index and potential for adverse effects. We assessed the clinical impacts of a linezolid TDM process and possible associations between patient demographics and treatment outcomes at a Veterans Affairs Hospital.

**Figure:**
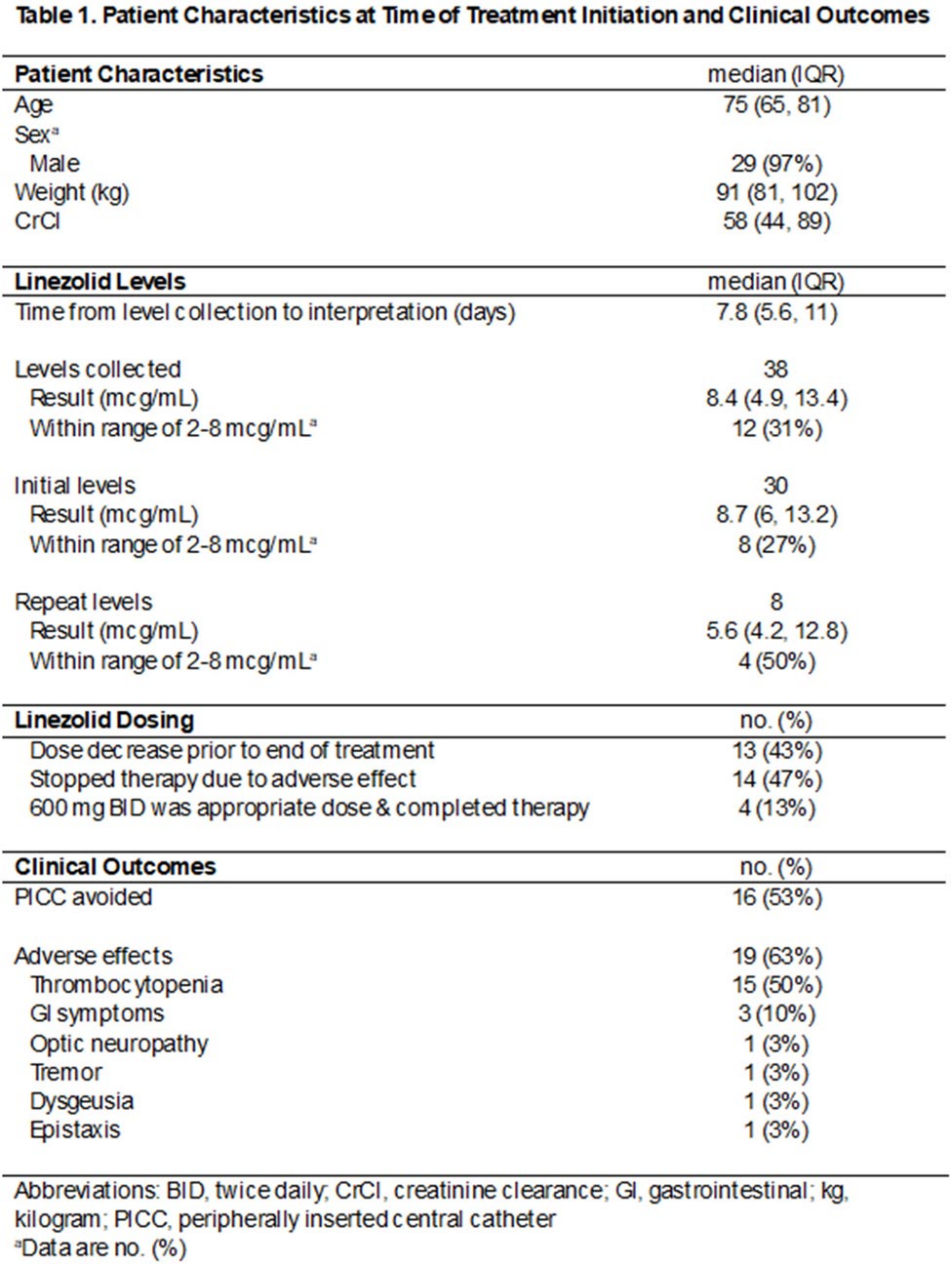

**Figure 1.**
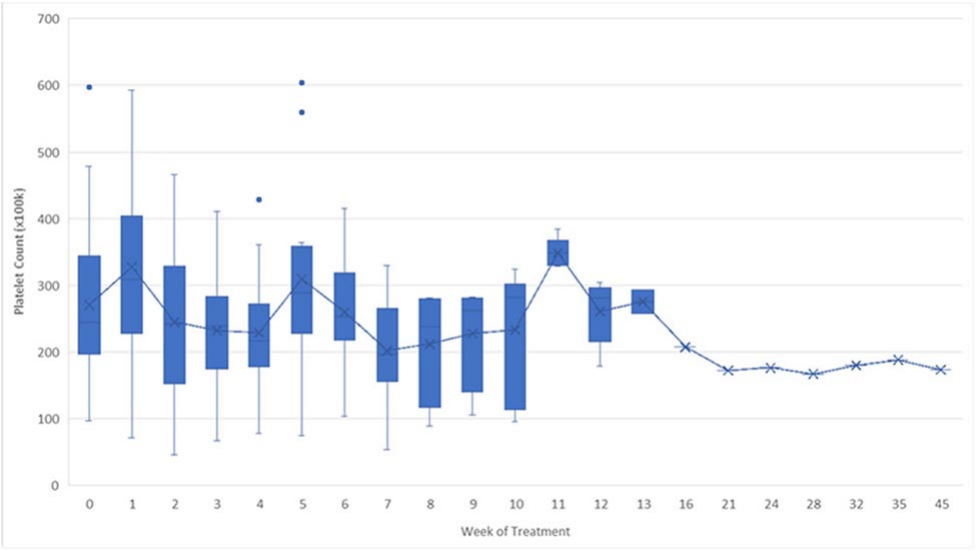
Platelet Count Over Time

**Methods:**

This was a single-center retrospective cohort study of patients with anticipated > 14-days of linezolid therapy and who had at least one serum trough level measured during their treatment course. Chart review occurred from January 2024 to December 2024. Data was collected on levels and changes to linezolid dosing, patient age, weight, renal function, WBC, platelet count, incidence of adverse effects, completion of intended duration of therapy, and peripherally inserted central catheter (PICC) avoidance.

**Results:**

A total of 30 encounters including 29 unique patients were captured. Thirty-eight linezolid levels were collected (30 initial, 8 repeat) with a median value of 8.4 mcg/mL. Twelve levels (31%) resulted within the therapeutic goal range of 2-8 mcg/mL. Thirteen patients (43%) required a dose decrease prior to the end of treatment, and 14 patients (47%) discontinued linezolid due to adverse effects. The most common adverse effect was thrombocytopenia (15 patients, 50%). Only 4 patients (13%) completed their intended linezolid treatment course at the FDA approved dose of 600 mg twice daily with a therapeutic level and without the need for a dose reduction. PICCs were avoided in 16 patients (53%).

**Conclusion:**

The majority of patients had linezolid trough levels above the therapeutic range when started on the standard FDA approved dose. TDM resulted in dose reductions and completion of the planned course in 13 of 30 patients, but thrombocytopenia was still common in the cohort. These findings suggest a need for additional studies to improve implementation of TDM and investigate alternative initial dosing in select patients.

**Disclosures:**

All Authors: No reported disclosures

